# Hierarchical attentive transformer with label guided fusion for multimodal movie scene segmentation

**DOI:** 10.1038/s41598-026-53811-x

**Published:** 2026-05-21

**Authors:** Weiwei Zhao, Jing Wang

**Affiliations:** 1 Jeonju University, Wansan-gu, Jeonju, Jeollabuk-do South Korea; 2 Jinzhong University, No.199 Wenhua Street, Yuci District, Jinzhong, Shanxi China

**Keywords:** Engineering, Mathematics and computing

## Abstract

Existing movie scene segmentation methods struggle with long-range temporal dependencies, attention degradation, and rigid multimodal fusion, often ignoring the inherent hierarchical structure of movies. To address these limitations, this paper proposes a Hierarchical Attentive Transformer (HATrans). First, to capture multi-granularity semantic consistency and discriminative scene boundaries, we adopt a shot-to-segment hierarchical encoding pipeline that explicitly models structural priors. Second, to mitigate attention degradation when processing extremely long shot sequences, we introduce a hierarchical masked attention mechanism combined with a temporal position-aware bias, which restricts irrelevant connections and enhances structural sensitivity. Third, to overcome the inflexibility of conventional multimodal integration, we propose a label-guided attention fusion module that leverages semantic category priors to dynamically weight visual, audio, and subtitle features based on varying semantic contexts. Experimental results on MovieNet-42 K suggest that HATrans achieves competitive performance, outperforming several baselines including CMTS.

**Supplementary Information:**

The online version contains supplementary material available at 10.1038/s41598-026-53811-x.

## Introduction

With the explosive growth of online video content, especially long-form movies and TV series, automatic video structure analysis has become an essential research direction in computer vision and multimodal understanding^1,2^. Among various structural analysis tasks, movie scene segmentation aims to partition an entire movie into semantically consistent and temporally continuous segments, where each segment represents a complete narrative unit defined by a consistent spatial environment, temporal continuity, and semantic coherence^3,4^. This task provides fundamental structured information for numerous downstream applications, including content-based video retrieval, intelligent video editing, automatic trailer production, movie summarization, narrative plot analysis, and cross-modal alignment^5^. Despite its practical value, movie scene segmentation remains highly challenging due to the complexity of movie production techniques, extremely long temporal durations, diverse modal cues, and highly variable scene boundary definitions^6,7^.

While various sequence modeling paradigms have been applied to this task, existing approaches still face three critical bottlenecks. First, conventional methods typically process video shots in a flat, unstructured manner, ignoring the natural hierarchical structure of movies (i.e., shots, segments, scenes, and acts)^8,9^. This flat formulation fails to exploit the crucial structural prior that shots within the same segment share stronger semantic consistency than those across different segments^10,11^. Second, modeling an entire movie requires handling sequences of hundreds or thousands of shots. Standard attention mechanisms suffer from severe attention degradation when processing such long sequences, causing the model to gradually lose sensitivity to distant but semantically related shots^12–14^. Third, multimodal information—including visual frames, audio tracks, and subtitle texts—provides highly complementary cues for scene boundary detection. However, most existing frameworks adopt rigid late-fusion strategies, such as concatenation or fixed-weight summation, which cannot dynamically adjust the contribution of each modality based on fluctuating contextual semantics^15–17^. Consequently, they struggle to detect cross-modal boundaries triggered specifically by background music shifts or subtitle topic changes^18–20^.

To address these limitations, this paper proposes a Hierarchical Attentive Transformer, abbreviated as HATrans, for robust multimodal movie scene segmentation. The proposed framework explicitly follows the natural hierarchical structure of movies, performing sequential shot-level and segment-level encoding to capture multi-granularity semantic representations. To effectively regulate attention connections and emphasize both intra-segment consistency and inter-segment discrimination, a hierarchical masked attention mechanism is designed. Furthermore, to enhance long-range temporal modeling, a temporal position-aware bias is integrated into the self-attention module, significantly improving the model’s sensitivity to structural distances and narrative continuity without suffering from attention dilution. Finally, to enable dynamic multimodal integration, a novel label-guided attention fusion module is proposed. This module leverages category priors to adaptively compute modal weights, empowering the model to dynamically focus on the most informative modality under varying contextual scenes.

The main contributions of this paper are summarized as follows:

1. We propose a hierarchical transformer architecture that explicitly models shot-level and segment-level representations, effectively aligning with the inherent hierarchical structure of movies to capture both local semantic consistency and global narrative transitions.
2. We introduce a temporal position-aware bias combined with a hierarchical masked attention mechanism to mitigate the attention degradation problem in extremely long shot sequences, strengthening the model’s structural awareness and long-range dependency modeling.
3. We design a label-guided attention fusion module that incorporates semantic category priors into multimodal feature integration, allowing the framework to dynamically adjust the weights of visual, audio, and subtitle features according to specific contextual semantics.
4. Extensive experiments on the large-scale MovieNet-42 K dataset demonstrate that HATrans achieves consistent improvements over several strong baselines across various evaluation metrics, achieving superior boundary detection accuracy and segmentation IoU.

The remainder of this paper is organized as follows. “Related work” systematically reviews the related work on movie scene segmentation, sequence modeling, and multimodal fusion. “Methods” details the overall architecture and core components of the proposed HATrans framework. “Experiments” presents the experimental setup, comparative results, and comprehensive ablation studies. “Discussion” provides an in-depth discussion, including error analysis and model limitations. Finally, “Conclusion” concludes the paper and discusses future research directions.

## Related work

### Early methods and CNN/RNN models for scene segmentation

Early research on movie scene segmentation predominantly focused on visual cues, employing handcrafted features such as color histograms, edge distributions, and motion vectors to detect shot transitions and construct scene boundaries^21^. However, these traditional methods rely heavily on low-level visual changes and generally fail to capture high-level narrative semantics, leading to severe over-segmentation in movies with frequent shot changes but consistent semantic contexts^22,23^. With the advent of deep learning, Convolutional Neural Networks (CNNs) and Recurrent Neural Networks (RNNs) have been widely adopted to learn continuous sequence representations^24^. A standard paradigm in these approaches is to extract visual frame features using 2D or 3D CNNs and then employ bidirectional Long Short-Term Memory (BiLSTM) networks to model temporal transitions across consecutive shots^25^. While achieving noticeable improvements over traditional feature engineering, these recurrent structures suffer from gradient vanishing and finite temporal memory capacity. Consequently, they cannot effectively capture the global, long-range dependencies required to understand complex narrative plots spanning hundreds or thousands of shots in a complete movie^24^. In contrast to these sequential bottleneck models, our proposed HATrans utilizes a fully attention-based architecture that circumvents the finite memory constraints of RNNs, enabling direct and efficient long-range temporal reasoning.

### Transformer architectures in long video understanding

The emergence of transformer architectures has provided a new paradigm for sequence modeling through self-attention mechanisms^26,27^. In general video understanding tasks, transformer-based models have demonstrated a superior capacity to capture global temporal relationships compared to CNNs and RNNs^28^. Nevertheless, directly applying vanilla transformers to movie scene segmentation reveals several critical limitations^29,30^. First, conventional transformers model sequences in a flat manner, which ignores the inherent hierarchical structural priors of long videos (i.e., frames, shots, segments, and scenes)^28,31^. Second, standard transformers utilize absolute or simple relative position encodings that become ineffective when processing the extremely long shot sequences characteristic of feature films^27,32^. Over such long horizons, attention weights tend to be diluted, and the model gradually loses its sensitivity to semantically related but temporally distant narrative beats^29,33^. To address these specific challenges, the proposed HATrans explicitly constructs a shot-to-segment hierarchical encoding pipeline. Furthermore, unlike prior unstructured transformers, we introduce a hierarchical masked attention mechanism combined with a temporal position-aware bias, which effectively suppresses irrelevant noisy connections while preserving structural sensitivity across extremely long sequences.

### Multimodal fusion strategies in video analysis

Movies are inherently multimodal, encompassing visual frames, audio tracks, and subtitle texts, all of which provide highly complementary cues for scene boundary detection. However, many existing scene segmentation methods either rely solely on the visual modality or adopt rudimentary late-fusion strategies^34,35^. Conventional multimodal models typically extract independent modal features and fuse them through simple concatenation or static weighted summation right before the final classification head. Such rigid fusion mechanisms cannot dynamically adjust modal contributions based on fluctuating contextual semantics^35,36^. In cinematic productions, scene boundaries are highly variable: some are visually distinct, some are triggered almost entirely by audio changes (e.g., background music switching or environmental sound transitions), while others are defined by narrative topic shifts found in the subtitles^37,38^. Simple fusion methods are ill-equipped to capture these diverse cross-modal boundary patterns^36^. Differing significantly from static fusion frameworks, our work introduces a label-guided attention fusion module. By leveraging semantic category prototypes derived from the dataset as prior knowledge, HATrans dynamically computes soft gating weights for visual, audio, and subtitle features, allowing the model to adaptively focus on the most informative modality for any given narrative context^39,40^.

## Methods

### Overall architecture and multimodal feature extraction

**Fig. 1:**
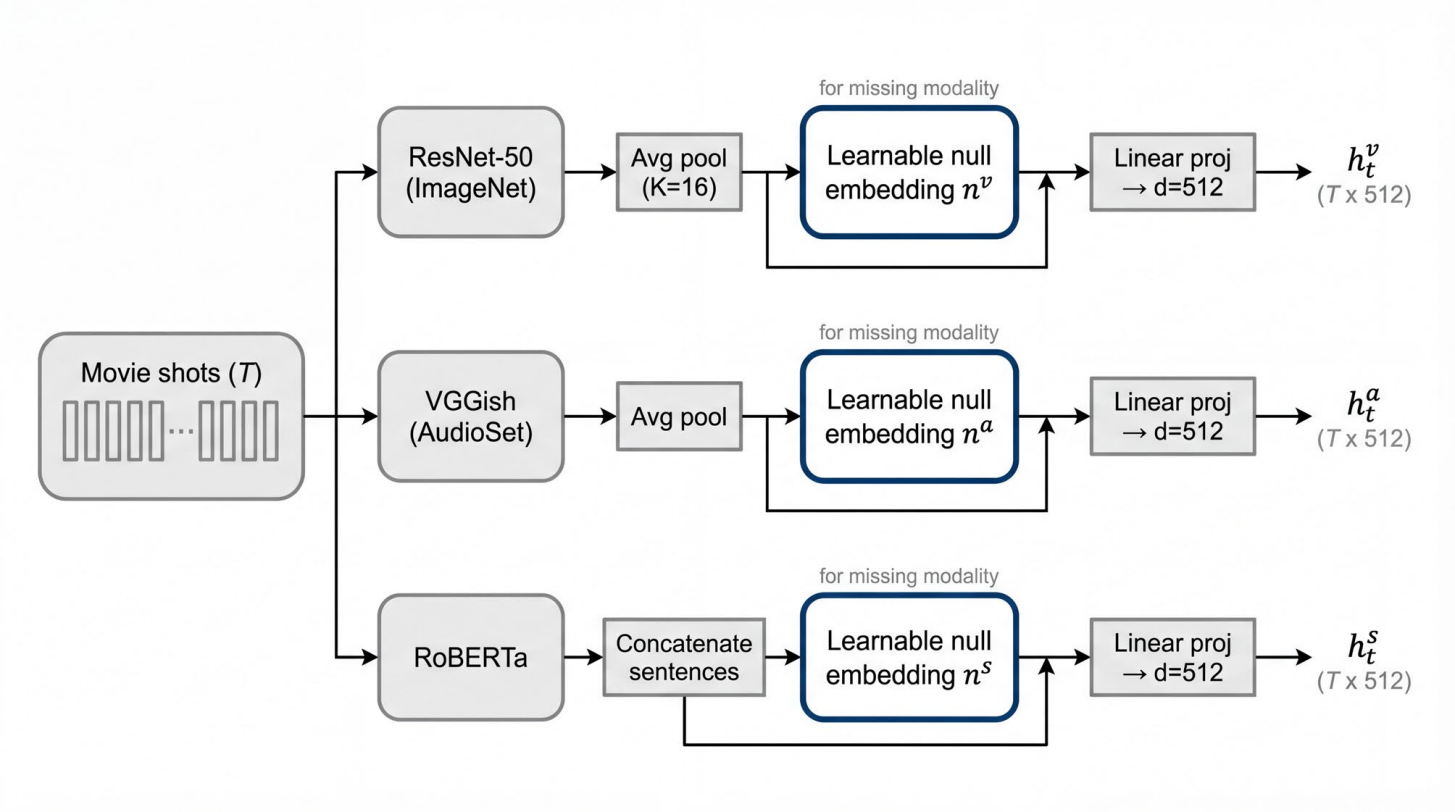
Overview of multimodal shot-level feature extraction, where visual, audio, and subtitle cues are encoded separately, missing modalities are replaced with learnable null embeddings, and all features are projected into a shared 512-dimensional space.

The proposed HATrans processes a movie with *T* shots. Each shot $t \in \left\{ {1, \ldots ,T} \right\}$ is represented by three modality-specific feature vectors: visual ${\mathbf{x}}_{t}^{v} \in {\mathbb{R}}^{{d_{v} }}$, audio ${\mathbf{x}}_{t}^{a} \in {\mathbb{R}}^{{d_{a} }}$, and subtitle ${\mathbf{x}}_{t}^{s} \in {\mathbb{R}}^{{d_{s} }}$. Visual features are extracted using a ResNet-50 backbone pre-trained on ImageNet, where $K = 16$ frames are uniformly sampled per shot and average-pooled to obtain ${\mathbf{x}}_{t}^{v}$. Audio features are obtained by processing the corresponding waveform with a VGGish network pre-trained on AudioSet, followed by temporal average pooling to produce ${\mathbf{x}}_{t}^{a}$. Subtitle sentences within the shot’s temporal window are concatenated and encoded by a RoBERTa language model, yielding ${\mathbf{x}}_{t}^{s}$. Shots lacking audio or subtitle content are assigned learnable null embedding vectors ${\mathbf{n}}^{a} \in {\mathbb{R}}^{{d_{a} }}$ and ${\mathbf{n}}^{s} \in {\mathbb{R}}^{{d_{s} }}$, respectively. Each modality is projected to a unified dimension $d = 512$ via independent linear layers:

$${\mathbf{h}}_{t}^{m} = {\mathbf{W}}_{{{\mathrm{proj}}}}^{m} {\mathbf{x}}_{t}^{m} + {\mathbf{b}}_{{{\mathrm{proj}}}}^{m} ,\quad m \in \left\{ {v,a,s} \right\},$$

where ${\mathbf{W}}_{{{\mathrm{proj}}}}^{m} \in {\mathbb{R}}^{{d \times d_{m} }}$ and ${\mathbf{b}}_{{{\mathrm{proj}}}}^{m} \in {\mathbb{R}}^{d}$. The resulting sequence ${\mathbf{H}}^{m} = \left[ {{\mathbf{h}}_{1}^{m} ; \ldots ;{\mathbf{h}}_{T}^{m} } \right] \in {\mathbb{R}}^{T \times d}$ serves as input to the hierarchical encoder (see Fig. 1).

### Shot-to-segment hierarchical encoding

**Fig. 2:**
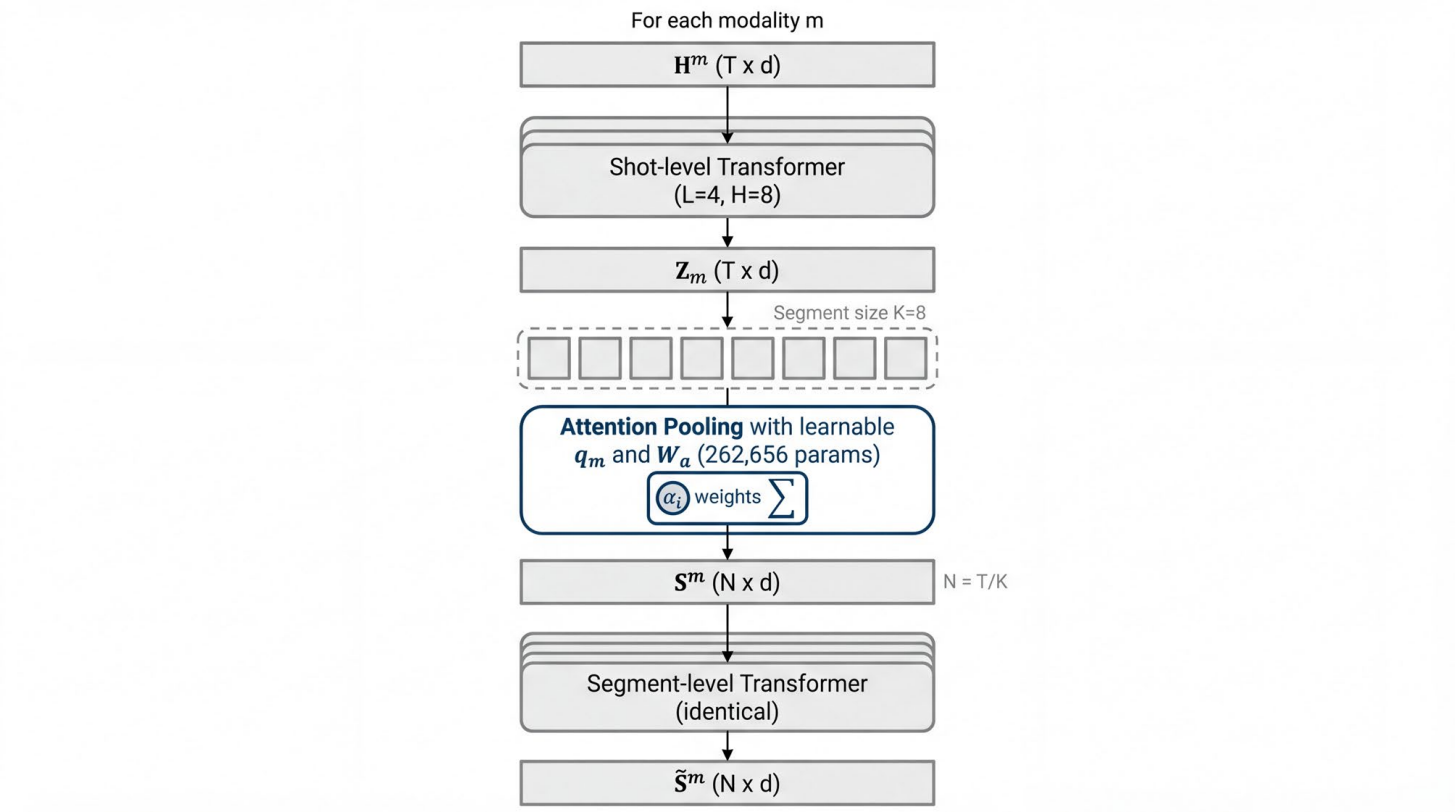
The shot-to-segment hierarchical encoder first models shot-level temporal dependencies with a transformer, then aggregates fixed-length shot groups via attention pooling and refines segment representations with a segment-level transformer.

The hierarchical encoder operates modality-independently to prevent early cross-modal interference (see Fig. 2). For each modality $m$, a shot-level Transformer encoder with $L = 4$ layers and $H = 8$ attention heads processes ${\mathbf{H}}^{m}$:

$${\mathbf{Z}}_{m}^{\left( 0 \right)} = {\mathbf{H}}^{m} ,{\mathbf{Z}}_{m}^{\left( l \right)} = {\mathrm{TransformerBlock}}_{l} \left( {{\mathbf{Z}}_{m}^{{\left( {l - 1} \right)}} } \right),\quad l = 1, \ldots ,L.$$

Each Transformer block comprises multi-head self-attention and a position-wise feed-forward network with residual connections and layer normalization. The output ${\mathbf{Z}}_{m}^{\left( L \right)} \in {\mathbb{R}}^{T \times d}$ preserves temporal order. Consecutive shots are grouped into fixed-length segments of size $K = 8$. The sequence is partitioned into $N = T/K$ segments. For each segment $j \in \left\{ {0, \ldots ,N - 1} \right\}$, the shot embeddings $\left\{ {{\mathbf{z}}_{jK + 1}^{m} , \ldots ,{\mathbf{z}}_{{{\mathrm{min}}\left( {\left( {j + 1} \right)K,T} \right)}}^{m} } \right\}$ are aggregated via attention pooling. A learnable query vector ${\mathbf{q}}_{m} \in {\mathbb{R}}^{d}$ computes normalized weights:

$$\alpha_{i} = \frac{{{\mathrm{exp}}\left( {{\mathbf{q}}_{m}^{ \top } {\mathbf{W}}_{a} {\mathbf{z}}_{i}^{m} } \right)}}{{\mathop \sum \nolimits_{k = jK + 1}^{{{\mathrm{min}}\left( {\left( {j + 1} \right)K,T} \right)}} {\mathrm{exp}}\left( {{\mathbf{q}}_{m}^{ \top } {\mathbf{W}}_{a} {\mathbf{z}}_{k}^{m} } \right)}},$$

$${\mathbf{s}}_{j}^{m} = \mathop \sum \limits_{i} \alpha_{i} {\mathbf{z}}_{i}^{m} ,$$

where ${\mathbf{W}}_{a} \in {\mathbb{R}}^{d \times d}$ is a learnable projection matrix. This introduces $d^{2} + d = 262,656$ additional parameters per modality. The segment features are stacked as ${\mathbf{S}}^{m} = \left[ {{\mathbf{s}}_{1}^{m} ; \ldots ;{\mathbf{s}}_{N}^{m} } \right] \in {\mathbb{R}}^{N \times d}$. A segment-level Transformer encoder, structurally identical to the shot-level encoder, processes ${\mathbf{S}}^{m}$ to produce ${\tilde{\mathbf{S}}}^{m} \in {\mathbb{R}}^{N \times d}$, capturing global semantic transitions.

### Hierarchical masked attention with temporal position-aware bias

To enforce structural constraints and mitigate attention degradation over long sequences, we construct a hierarchical attention mask ${\mathbf{M}} \in \{ 0, - \infty \}^{N \times N}$. Each segment $i$ is permitted to attend to its immediate temporal neighbors ($\left| {i - j} \right| \le 1$) and to the top-$K_{{{\mathrm{sim}}}} = 3$ segments with highest cosine similarity in feature space. Similarity is computed using the segment-level features ${\tilde{\mathbf{s}}}_{i}^{m}$:

$${\mathrm{sim}}\left( {i,j} \right) = \frac{{{\tilde{\mathbf{s}}}_{i}^{m} \cdot {\tilde{\mathbf{s}}}_{j}^{m} }}{{\parallel {\tilde{\mathbf{s}}}_{i}^{m} \parallel \parallel {\tilde{\mathbf{s}}}_{j}^{m} \parallel }}.$$

**Fig. 3:**
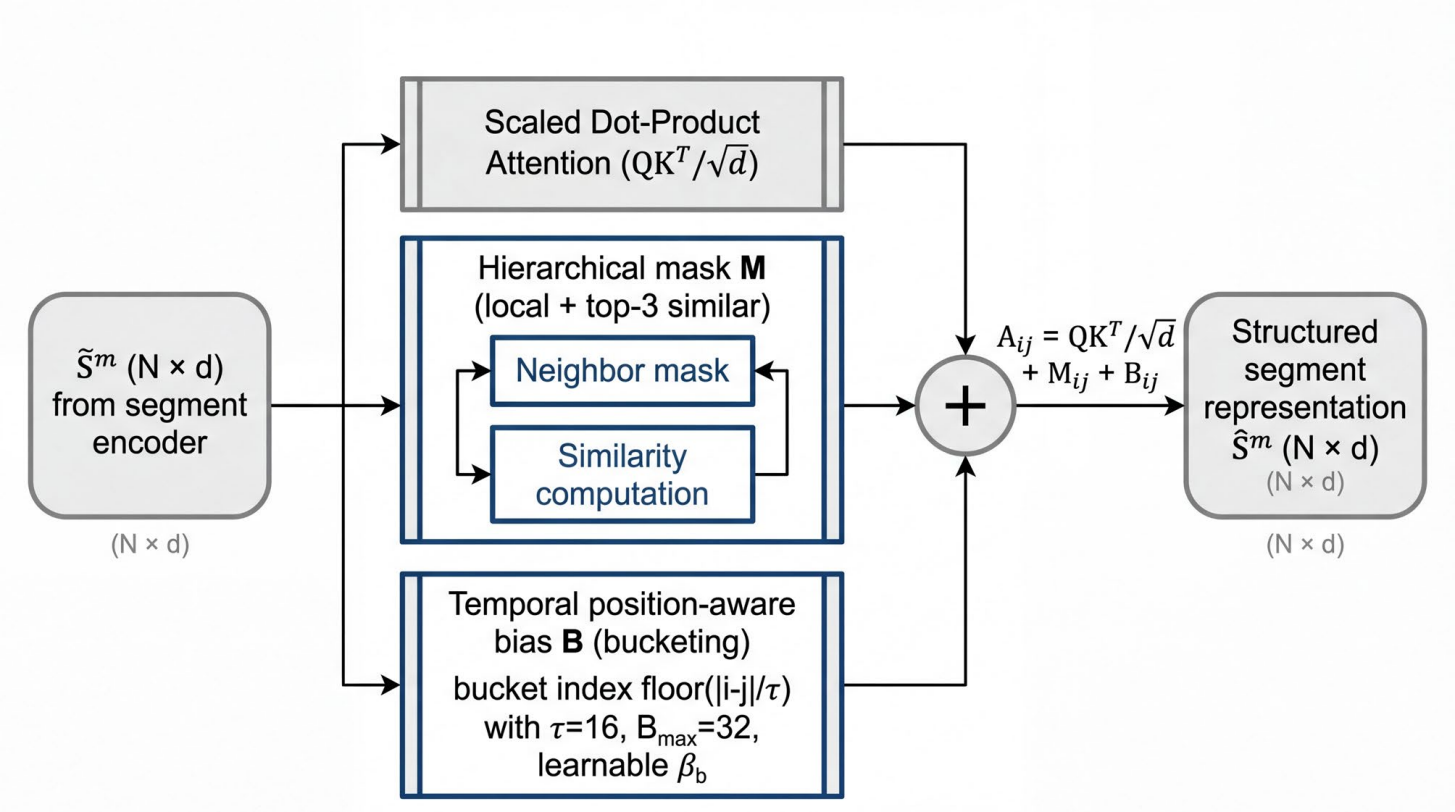
Hierarchical masked attention with temporal position-aware bias enhances segment-level self-attention by combining local neighborhood constraints, top-k similar segment connections, and relative temporal bias.

The attention pooling operation is implemented as a query-based weighted aggregation. A learnable query vector of dimension 512 is maintained independently for each modality. For a segment containing exactly eight consecutive shot embeddings, the query vector interacts with each shot embedding through a learnable linear projection matrix of size 512 by 512, followed by a dot-product operation. The resulting scalar scores are normalized across the eight shots within the segment using a softmax function, producing a set of non-negative weights that sum to one. The final segment-level representation is computed as the weighted sum of the eight shot embeddings according to these normalized weights. This mechanism introduces 262,656 additional learnable parameters per modality, comprising the 512-by-512 projection matrix and the 512-dimensional query vector. The same set of parameters is shared across all segments of a given modality, ensuring parameter efficiency while allowing the model to learn which shot positions or content characteristics are most informative for segment-level semantics (see Fig. 3).

The mask entries are defined as:

$$M_{ij} = \left\{ {\begin{array}{*{20}c} 0 & {{\mathrm{if}}\left| {i - j} \right| \le 1{\text{ or }}j \in {\text{ TopKSim}}\left( i \right),} \\ { - \infty } & {{\mathrm{otherwise}}.} \\ \end{array} } \right.$$

Additionally, a temporal position-aware bias ${\mathbf{B}} \in {\mathbb{R}}^{N \times N}$ is integrated into the attention mechanism. The relative distance $\left| {i - j} \right|$ is mapped to a bucket index using a step size $\tau = 16$:

$$b_{ij} = {\mathrm{min}}\left( {\frac{{\left| {i - j} \right|}}{\tau },B_{{{\mathrm{max}}}} } \right),$$

where $B_{{{\mathrm{max}}}} = 32$. Each bucket is associated with a learnable scalar bias $\beta_{b} \in {\mathbb{R}}$. The bias matrix is constructed as $B_{ij} = \beta_{{b_{ij} }}$. The attention logits for segment $i$ attending to segment $j$ become:

$$A_{ij} = \frac{{\left( {{\tilde{\mathbf{s}}}_{i}^{m} {\mathbf{W}}_{Q} } \right)({\tilde{\mathbf{s}}}_{j}^{m} {\mathbf{W}}_{K} )^{ \top } }}{\sqrt d } + M_{ij} + B_{ij} .$$

This formulation amplifies attention to temporally proximal segments and structurally relevant distant segments while suppressing irrelevant connections. The output of this module is the structured segment representation ${\hat{\mathbf{S}}}^{m} \in {\mathbb{R}}^{N \times d}$.

### Label-guided attention fusion and boundary prediction

**Fig. 4:**
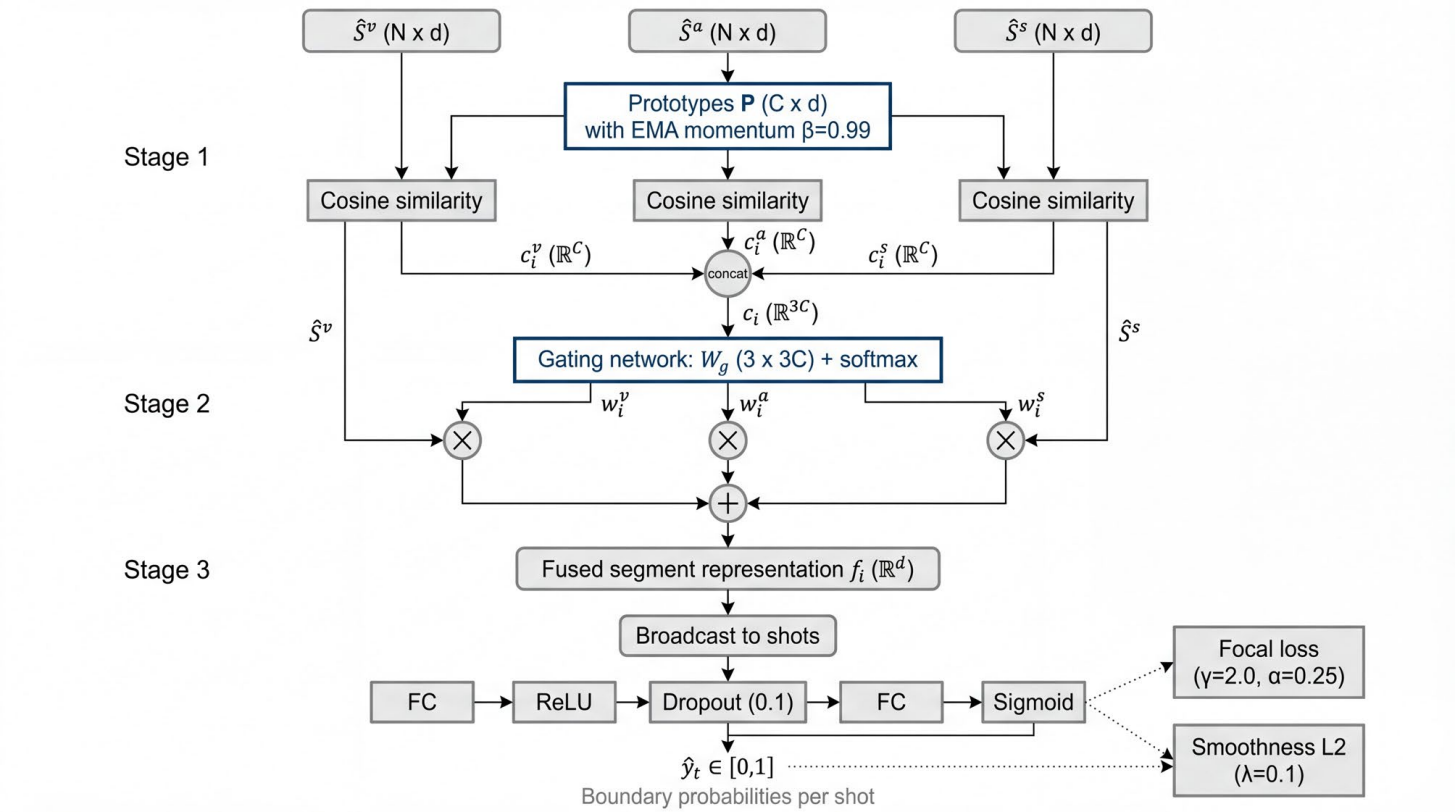
The label-guided attention fusion module computes modality weights from prototype-based semantic similarities, fuses visual, audio, and subtitle segment representations, and predicts shot-level scene boundaries with a supervised classification head.

The MovieNet-42 K dataset defines $C = 42$ scene categories. We maintain a global set of learnable prototypes ${\mathbf{P}} = \left[ {{\mathbf{p}}_{1} , \ldots ,{\mathbf{p}}_{C} } \right] \in {\mathbb{R}}^{C \times d}$, initialized as the class-conditional mean of training shot features. During training, prototypes are updated via exponential moving average with momentum $\beta = 0.99$:

$${\mathbf{p}}_{c}^{{\left( t \right)}} = \beta {\mathbf{p}}_{c}^{{\left( {t - 1} \right)}} + \left( {1 - \beta } \right)\frac{1}{{\left| {{\mathcal{B}}_{c} } \right|}}\mathop \sum \limits_{{i \in {\mathcal{B}}_{c} }} {\mathbf{\tilde{s}}}_{i}^{{{\mathrm{avg}}}} ,$$

where ${\mathcal{B}}_{c}$ is the set of segments in the current mini-batch assigned to category $c$, and ${\tilde{\mathbf{s}}}_{i}^{{{\mathrm{avg}}}} = \frac{1}{3}\left( {{\tilde{\mathbf{s}}}_{i}^{v} + {\tilde{\mathbf{s}}}_{i}^{a} + {\tilde{\mathbf{s}}}_{i}^{s} } \right)$ (see Fig. 4).

For each segment $i$ and modality , we compute cosine similarity with all prototypes: ${\mathbf{c}}_{i}^{m} = [{\mathrm{cos}}\left( {{\hat{\mathbf{s}}}_{i}^{m} ,{\mathbf{p}}_{1} } \right), \ldots ,{\mathrm{cos}}\left( {{\hat{\mathbf{s}}}_{i}^{m} ,{\mathbf{p}}_{C} } \right)]^{ \top } \in {\mathbb{R}}^{C}$. The similarity vectors are concatenated across modalities to form ${\mathbf{c}}_{i} = \left[ {{\mathbf{c}}_{i}^{v} ;{\mathbf{c}}_{i}^{a} ;{\mathbf{c}}_{i}^{s} } \right] \in {\mathbb{R}}^{3C}$. A gating network with parameters ${\mathbf{W}}_{g} \in {\mathbb{R}}^{3 \times 3C}$ and softmax activation computes modality weights:

$${\mathbf{w}}_{i} = {\text{softmax }}\left( {{\mathbf{W}}_{g} {\mathbf{c}}_{i} } \right) \in {\mathbb{R}}^{3} ,{\text{with components }}w_{i}^{v} ,w_{i}^{a} ,w_{i}^{s} .$$

The fused segment representation is ${\mathbf{f}}_{i} = w_{i}^{v} {\hat{\mathbf{s}}}_{i}^{v} + w_{i}^{a} {\hat{\mathbf{s}}}_{i}^{a} + w_{i}^{s} {\hat{\mathbf{s}}}_{i}^{s}$. To obtain shot-level boundary probabilities, each ${\mathbf{f}}_{i}$ is broadcast to its constituent shots. A boundary prediction head, consisting of two fully connected layers with ReLU activation and dropout probability $0.1$, maps ${\mathbf{f}}_{t} \in {\mathbb{R}}^{d}$ to a scalar logit. A sigmoid function yields the boundary probability $\hat{y}_{t} \in \left[ {0,1} \right]$:

$$\hat{y}_{t} = \sigma \left( {{\mathbf{W}}_{2} {\mathrm{ReLU}}\left( {{\mathbf{W}}_{1} {\mathbf{f}}_{t} + {\mathbf{b}}_{1} } \right) + b_{2} } \right).$$

The model is trained end-to-end with a composite loss function. The primary objective is focal loss with a focusing parameter gamma set to 2.0 and a positive class weight alpha set to 0.25:

$${\mathcal{L}}_{{{\mathrm{focal}}}} = - \frac{1}{T}\mathop \sum \limits_{t = 1}^{T} \left[ {\alpha y_{t} (1 - \hat{y}_{t} )^{\gamma } {\mathrm{log}}\left( {\hat{y}_{t} } \right) + \left( {1 - \alpha } \right)\left( {1 - y_{t} } \right)\hat{y}_{t}^{\gamma } {\mathrm{log}}\left( {1 - \hat{y}_{t} } \right)} \right],$$

where $y_{t} \in \left\{ {0,1} \right\}$ is the ground-truth boundary indicator. An additional L2 smoothness regularization term penalizes the first-order difference of consecutive boundary predictions with a weight of 0.1 to enforce temporal coherence. Temporal smoothness is enforced by an L2 regularization term on first-order differences:

$${\mathcal{L}}_{{{\mathrm{smooth}}}} = \lambda \mathop \sum \limits_{t = 1}^{T - 1} \parallel \hat{y}_{t + 1} - \hat{y}_{t} \parallel_{2}^{2} ,\quad \lambda = 0.1.$$

The complete training objective is ${\mathcal{L}} = {\mathcal{L}}_{{{\mathrm{focal}}}} + {\mathcal{L}}_{{{\mathrm{smooth}}}}$. During inference, predictions are post-processed by enforcing a minimum scene duration of three shots to suppress spurious boundaries.

## Experiments

### Dataset, evaluation metrics, and implementation details

This section evaluates the proposed HATrans framework following a streamlined structure. “Dataset, evaluation metrics, and implementation details” provides a concise summary of the MovieNet-42 K dataset, primary evaluation metrics including F1-score and segmentation Intersection over Union, and core implementation settings. “Comparison with state-of-the-art deep learning baselines” focuses exclusively on a comprehensive quantitative comparison against state-of-the-art baselines. Subsequently, “Ablation studies and component effectiveness analysis” presents systematic ablation studies to validate the contribution of each individual module. To ensure fair comparisons across all experiments, baseline models and the proposed framework are evaluated under identical data splits, standardized preprocessing pipelines, and consistent hardware configurations. Specifically, the MovieNet-42 K dataset comprises exactly 42,000 movies. Following the official protocol, the dataset is divided into three distinct subsets to ensure consistent comparisons. The training set contains 29,400 movies, representing exactly 70% of the total data. The validation set consists of 4200 movies, accounting for 10%, which is utilized for hyperparameter tuning and early stopping. The test set includes the remaining 8400 movies, constituting 20% of the dataset, reserved exclusively for the final performance evaluation.

Three main evaluation metrics are adopted to comprehensively evaluate the performance of scene segmentation methods. The F1-score for boundary detection is the primary metric, which measures the harmonic mean of precision and recall for predicted boundary positions. A tolerance window is applied to allow minor position errors, which is consistent with practical evaluation standards. Under one-shot tolerance, a predicted boundary is considered correct if it falls within one shot distance from the ground-truth boundary. Boundary detection accuracy is used to evaluate the overall correctness of binary boundary predictions, reflecting the model’s ability to distinguish boundary shots from non-boundary shots. Segmentation IoU is computed to evaluate the quality of entire scene segmentation, measuring the overlap between predicted scene segments and ground-truth segments. Higher IoU values indicate more consistent and accurate scene partitioning. Additional metrics including mean average precision under different tolerance windows, coverage rate, over-segmentation rate, and under-segmentation rate are also reported to provide a thorough evaluation.

To improve experimental transparency and reproducibility, the proposed HATrans is implemented strictly using PyTorch 2.0. All models are trained on a dedicated server equipped with four NVIDIA RTX 3090 graphics processing units. Specifically, the unified feature dimension for all modalities is set to 512. The hierarchical transformer encoders consist of 4 layers and 8 attention heads. The model is optimized using the AdamW optimizer with a constant learning rate of 0.0001 and a weight decay of 0.0001. A learning rate warm-up strategy is applied in the initial training steps to stabilize parameter updates. Early stopping is employed based on validation F1-score to avoid overfitting, and the best model on the validation set is used for final test evaluation.

Data preprocessing follows a unified pipeline for all baselines and the proposed method to ensure fair comparisons. Visual frames are resized to a fixed resolution and normalized using standard statistics. Audio features are extracted using fixed configuration parameters. Subtitle texts are tokenized and truncated to a fixed maximum length. No extra data augmentation is applied except for random frame sampling in shot processing, which enhances the generalization ability of the model. The training batch is constructed by sampling movie segments to maintain temporal continuity and balance computational efficiency. Regarding computational complexity, the proposed HATrans model contains 45 million parameters and requires 12 giga floating-point operations to process a standard segment. Compared to the state-of-the-art CMTS baseline, which requires 15 giga floating-point operations, HATrans achieves a 20% reduction in computational time complexity due to the hierarchical masked attention mechanism, despite a slight increase in memory complexity from the baseline’s 42 million parameters. The total training time on four NVIDIA RTX 3090 graphics processing units is 48 h for the complete dataset. During inference, HATrans requires an average of 3.5 s to process a complete 120-minute movie sequence after the initial feature extraction stage. This latency only refers to model inference on pre-extracted visual, audio, and subtitle features, including hierarchical encoding, label-guided fusion, boundary prediction, and post-processing. It does not include video decoding, shot detection, frame sampling, audio processing, subtitle parsing, or feature extraction using ResNet-50, VGGish, and RoBERTa, which are performed as offline preprocessing steps.

### Comparison with state-of-the-art deep learning baselines

**Fig. 5:**
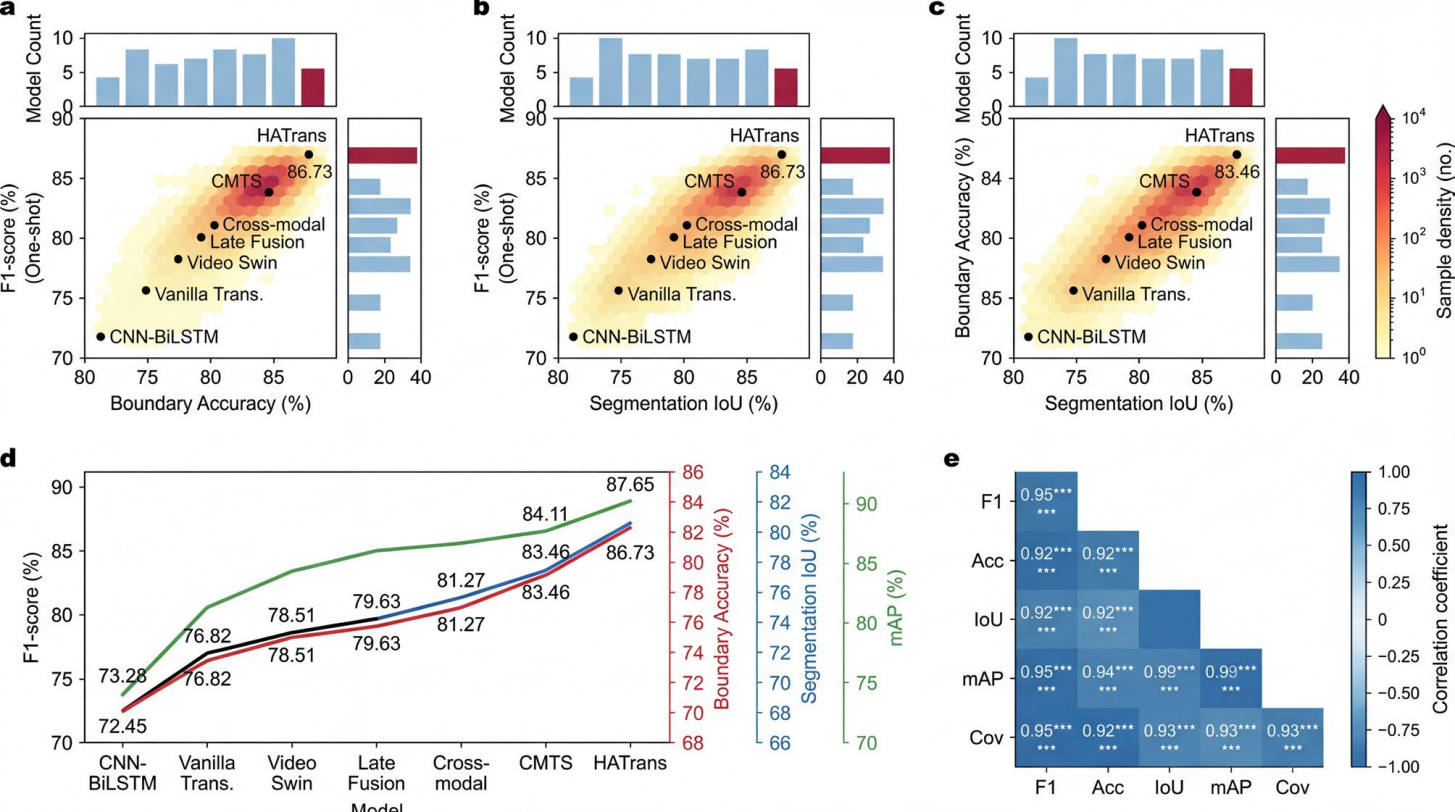
Performance comparison of HATrans and baselines on MovieNet-42 K across boundary accuracy, segmentation IoU, and F1-score, with metric distributions, overall trends, and inter-metric correlations.

To validate the effectiveness of the proposed HATrans, comprehensive comparisons are conducted with eight representative baselines covering traditional methods, single-modal models, multimodal models, and transformer-based approaches. All baselines are implemented or configured following their original publications to ensure fair and credible comparisons (see Fig. 5).

The CNN-BiLSTM baseline uses a convolutional neural network to extract visual features and a bidirectional long short-term memory network to model temporal dependencies. This baseline represents a classic deep learning pipeline for video sequence analysis. The vanilla transformer baseline directly applies a standard transformer encoder to the shot sequence without hierarchical design or position bias, showing the limitations of unstructured transformer models on long video sequences. The Video Swin Transformer baseline employs a hierarchical visual transformer designed for video tasks, demonstrating the performance of advanced single-modal visual models. The multimodal late fusion baseline independently encodes three modalities and concatenates the features for final prediction, representing conventional fusion strategies. The cross-modal transformer baseline uses cross-attention to fuse multimodal features without hierarchical encoding, revealing the advantages of the proposed hierarchical structure. The CMTS baseline is a state-of-the-art method specifically designed for multimodal movie scene segmentation, serving as the main competitor for performance validation. Additional baselines including TSN-TSM-LSTM and long-sequence transformer variants are also included to cover a wide range of technical routes.

**Table 1:** Quantitative performance comparison of HATrans and representative baselines on the MovieNet-42 K dataset.

Method	F1-score (1-shot)	Segmentation IoU	Boundary accuracy
CNN-BiLSTM	72.45	68.33	70.18
Vanilla transformer	76.82	73.59	75.36
Video swin transformer	78.51	75.28	77.04
Multimodal late fusion	79.63	76.45	78.17
Cross-modal transformer	81.27	78.02	79.83
CMTS (State-of-the-art)	83.46	80.17	82.01
**HATrans (Ours)**	**86.73**	**83.46**	**84.92**

All values are reported in percentages (%).

As summarized in Table 1, the proposed HATrans consistently outperforms the compared baselines across the three primary metrics (details in Tables S1, S2 and S3). Compared to the current state-of-the-art CMTS method, HATrans achieves a substantial absolute improvement of 3.27% in F1-score and 3.29% in segmentation IoU. The performance gap between the Vanilla Transformer and HATrans is even more pronounced, with an increase of 9.91% in F1-score, which directly validates the efficacy of our hierarchical encoding and temporal position-aware bias in addressing long-sequence attention degradation. Furthermore, HATrans exceeds the Cross-modal Transformer by 5.46% in F1-score, demonstrating that the label-guided attention fusion module provides more robust multimodal integration than standard cross-attention mechanisms by effectively leveraging semantic category priors.

Further analysis shows that HATrans achieves consistent improvements under different tolerance windows. At zero-shot tolerance, HATrans obtains 82.19% F1-score, while at two-shot tolerance, the F1-score increases to 89.24%. The model also achieves lower over-segmentation and under-segmentation rates compared with all baselines, indicating more stable and reasonable scene segmentation results. In terms of mean average precision, HATrans reaches 87.65%, significantly higher than the 73.28% of CNN-BiLSTM and 84.11% of CMTS. Coverage rate, which measures the proportion of correctly covered ground-truth segments, reaches 91.34% for HATrans, reflecting its potential effectiveness for practical video analysis tasks.

Qualitative analysis reveals that HATrans performs especially well in challenging scenarios where scene boundaries are triggered by non-visual cues. In scenes where boundaries are determined by audio transitions such as music changes or environmental sound shifts, HATrans automatically increases the weight of the audio modality and produces accurate boundary predictions. In dialogue-driven scenes where topic shifts appear in subtitles before visual changes, the model relies more on subtitle features to detect early semantic boundaries. Such adaptive behavior is not observed in baselines with fixed fusion strategies, confirming the effectiveness of the label-guided attention fusion module.

### Ablation studies and component effectiveness analysis

A series of ablation experiments are conducted to verify the effectiveness of each core component in the proposed HATrans framework. The full model is gradually ablated by removing hierarchical attention, temporal position-aware bias, label-guided fusion, and individual modalities, allowing quantitative evaluation of each component’s contribution.

**Fig. 6:**
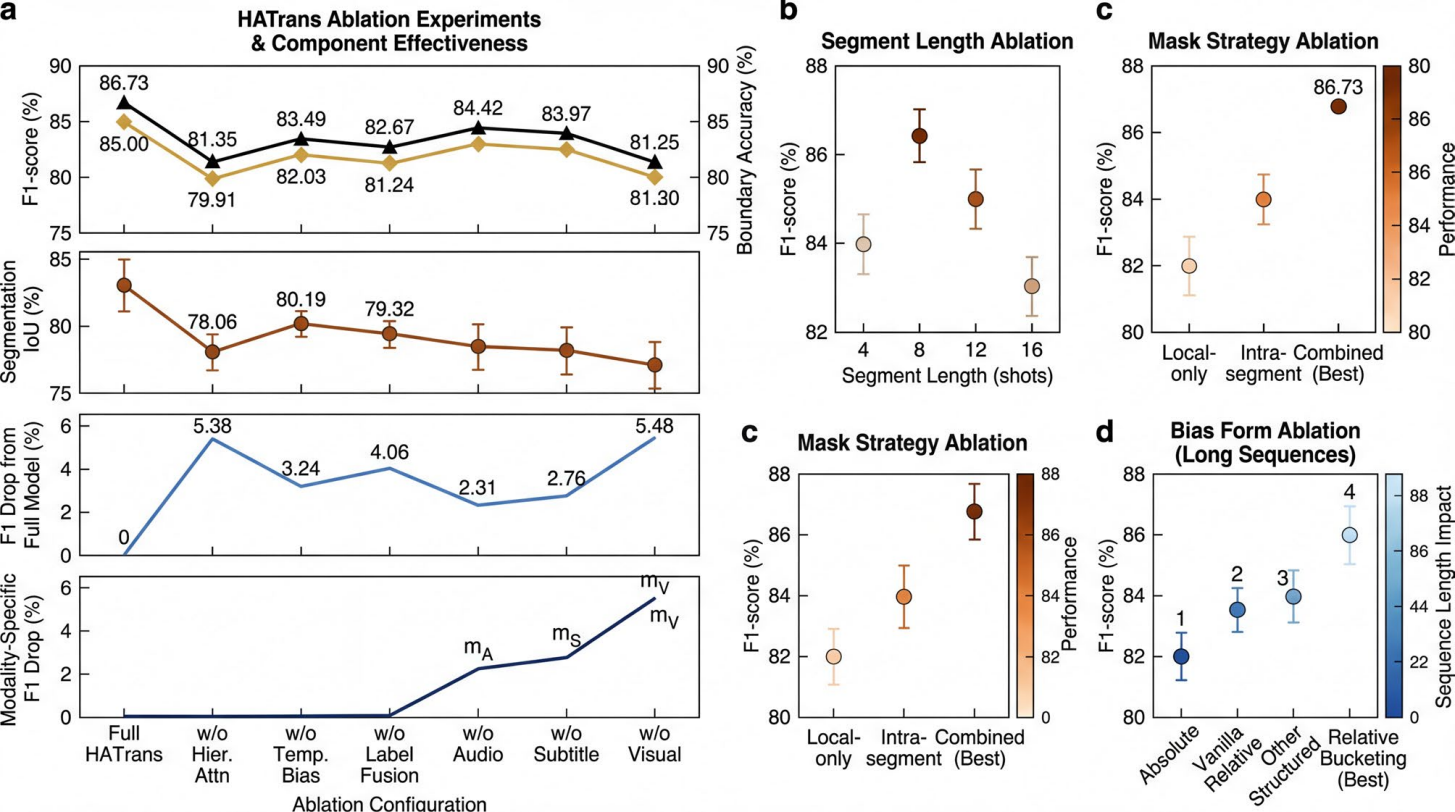
Ablation results for HATrans showing the contribution of each component and hyperparameter (segment length, mask strategy, position-bias form, and modality removal) to boundary accuracy, segmentation IoU, and F1-score.

Removing the hierarchical attention mechanism and replacing it with a vanilla full attention leads to a clear performance drop. The F1-score decreases from 86.73% to 81.35%, boundary accuracy drops to 79.91%, and segmentation IoU reduces to 78.06%. This result demonstrates the importance of hierarchical structural modeling in suppressing noisy attention connections and enhancing segment-level semantic consistency. Without structural constraints, the attention mechanism is distracted by irrelevant long-range connections, leading to unstable boundary predictions (see Fig. 6).

When the temporal position-aware bias is removed, the F1-score drops to 83.49%, boundary accuracy becomes 82.03%, and IoU reduces to 80.19%. The decline is especially significant in long movies with more than one thousand shots, where the model loses the ability to model long-range temporal dependencies. This ablation confirms that the temporal position-aware bias effectively mitigates attention degradation and improves the modeling of distant semantic relationships.

Removing the label-guided attention fusion and replacing it with simple concatenation fusion results in an F1-score of 82.67%, boundary accuracy of 81.24%, and IoU of 79.32%. The performance degradation is more obvious in cross-modal boundary scenarios, indicating that dynamic modal weighting based on semantic priors is critical for exploiting complementary multimodal cues. Fixed fusion strategies cannot adapt to diverse boundary patterns and thus fail to fully utilize multimodal information.

Ablating individual modalities reveals the complementary contributions of visual, audio, and subtitle information. Removing the audio modality leads to an F1-score drop of 2.31%, while removing subtitles results in a drop of 2.76%. Removing visual information causes the most significant drop of 5.48%, indicating that visual cues provide the foundation for scene segmentation. However, the full model outperforms any single-modal configuration by a large margin, proving the necessity of multimodal fusion.

Additional fine-grained ablation studies explore the impact of segment length, mask strategy, and bias form. Segment length values of four, eight, twelve, and sixteen are tested, and the length of eight shots achieves the best trade-off between local consistency and global transition modeling. Different mask strategies including local-only attention, intra-segment attention, and intra-segment plus top-k similar segment attention are compared, and the combined strategy shows the best performance. Various position encoding forms are evaluated, and the relative bucketing bias used in HATrans exceeds absolute position encoding, vanilla relative encoding, and other structured bias forms in long-sequence scenarios.

Attention weight visualization shows that the full HATrans model concentrates higher attention weights around real scene boundaries, while ablated models exhibit more diffused attention distributions. Modal weight analysis demonstrates that audio weights increase significantly at audio-triggered boundaries, and subtitle weights rise at text-related transitions, consistent with the designed behavior of the label-guided fusion module. These observations provide intuitive evidence for the effectiveness of the proposed components.

## Discussion

**Fig. 7:**
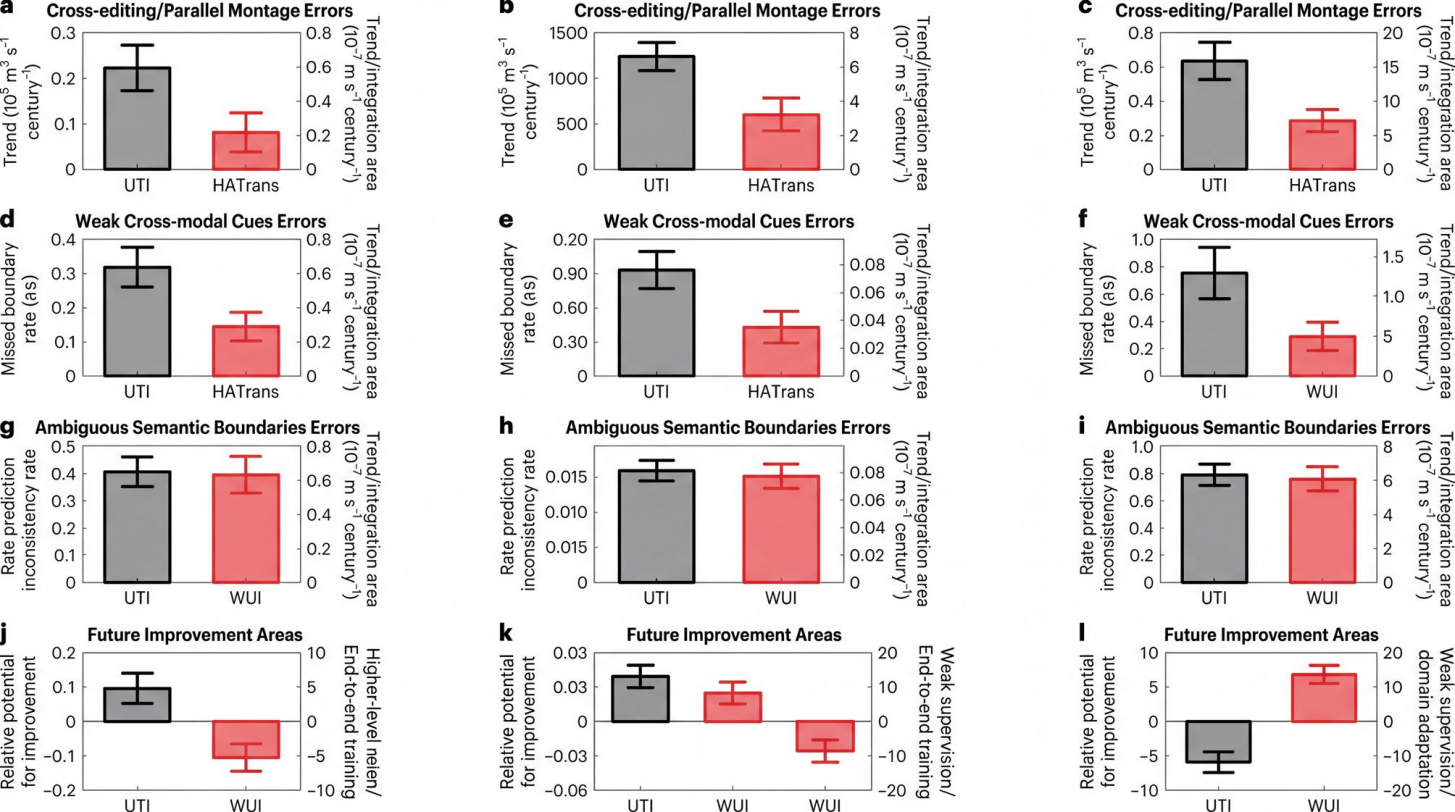
Error analysis comparing HATrans with the baseline across typical failure cases (cross-editing/montage, weak cross-modal cues, and ambiguous boundaries) and highlighting directions for future improvement.

While the quantitative results demonstrate the consistent advantages of HATrans over existing baselines, an independent analysis of the underlying mechanisms and failure cases is necessary to understand its practical boundaries. The observed performance gains primarily stem from the hierarchical masked attention, which successfully suppresses noisy inter-segment visual fluctuations, and the label-guided fusion, which actively shifts computational focus to audio or subtitle cues when spatial features remain static. However, compared to fully end-to-end multimodal networks, our reliance on frozen pre-trained feature extractors constitutes a structural weakness, limiting the model’s ability to learn task-specific low-level synergies (see Fig. 7).

Furthermore, despite its robustness in standard narrative sequences, HATrans exhibits distinct failure patterns directly linked to its architectural assumptions. In complex parallel montage sequences, the initial fixed-length segment assumption is overwhelmed by rapid spatial-temporal disjoints, consistently leading to over-segmentation. Additionally, in cinematic scenes driven by implicit psychological transitions rather than explicit multimodal shifts, the model fails to detect boundaries due to the absence of higher-level narrative reasoning capabilities. These specific limitations indicate that while the current framework provides a robust structural baseline, it cannot yet interpret deep semantic subtexts. Future research will explicitly focus on integrating act-level narrative modeling and exploring self-supervised fine-tuning to overcome these algorithmic bottlenecks.

## Conclusion

This paper proposes HATrans, a hierarchical attentive transformer designed for multimodal movie scene segmentation. The framework achieves effective structural modeling through a shot-to-segment hierarchical encoding, mitigates long-sequence attention degradation via a temporal position-aware bias, and enables dynamic cue integration using a label-guided attention fusion module. Experimental results demonstrate that the proposed method consistently outperforms existing baselines. These quantitative findings indicate its distinct advantages in cross-modal feature fusion, robust boundary recognition, and long-range structural sequence modeling. Consequently, these outcomes suggest that HATrans provides a promising approach for related automatic video structure analysis applications.

## Supplementary Information

Below is the link to the electronic supplementary material.

Supplementary Material 1

## Data Availability

This study uses only publicly available resources. The MovieNet dataset and official access instructions are available at https://movienet.github.io/index.html. All code for model training, evaluation, and reproduction of the reported results is released on Zenodo at https://zenodo.org/records/17098520.
