# Supplementary material for: Hierarchical attentive transformer with label guided fusion for multimodal movie scene segmentation
Source: Sci Rep. 2026 May 21;16:23191. doi: 10.1038/s41598-026-53811-x (PMC13396173; doi:10.1038/s41598-026-53811-x)
Supplement: Supplementary file 1 — Supplementary Material 1 [file 41598_2026_53811_MOESM1_ESM.docx]

**Table S1. Ablation study of core components in HATrans on MovieNet-42K.**

| **Model Variant** | **F1-score (1-shot)** | **Segmentation IoU** | **Boundary Accuracy** |
| --- | --- | --- | --- |
| Full HATrans | 86.73 | 83.46 | 84.92 |
| w/o Hierarchical Masked Attention | 81.35 | 78.06 | 79.91 |
| w/o Temporal Position-Aware Bias | 83.49 | 80.19 | 82.03 |
| w/o Label-Guided Fusion | 82.67 | 79.32 | 81.24 |
| w/o Audio Modality | 84.42 | 81.15 | 82.61 |
| w/o Subtitle Modality | 83.97 | 80.70 | 82.19 |
| w/o Visual Modality (Audio+Subtitle only) | 81.25 | 77.98 | 79.44 |

**Table S2. Sensitivity analysis of key hyperparameters on validation set F1-score.**

| **Hyperparameter** | **Value** | **F1-score (1-shot)** |
| --- | --- | --- |
| Segment Length K | 4 | 84.91 |
|  | 8 | 86.73 |
|  | 12 | 86.02 |
|  | 16 | 84.38 |
| Top-k Similarity Segments | 1 | 85.24 |
|  | 3 | 86.73 |
|  | 5 | 86.15 |
| Position Bias Bucket Size τ | 8 | 85.67 |
|  | 16 | 86.73 |
|  | 32 | 85.98 |

**Table S3. Comparison of multimodal fusion strategies on MovieNet-42K.**

| **Fusion Strategy** | **F1-score (1-shot)** | **Segmentation IoU** | **Boundary Accuracy** |
| --- | --- | --- | --- |
| Visual Only | 79.42 | 76.18 | 77.53 |
| Audio Only | 68.75 | 64.92 | 66.81 |
| Subtitle Only | 65.38 | 61.54 | 63.27 |
| Late Concatenation | 82.67 | 79.32 | 81.24 |
| Fixed-Weight Summation | 83.01 | 79.85 | 81.76 |
| Cross-Modal Attention | 83.46 | 80.17 | 82.01 |
| Label-Guided Fusion (Ours) | 86.73 | 83.46 | 84.92 |
